# Supplementary material for: Reconstructing the electrical structure of dust storms from locally observed electric field data
Source: Nat Commun. 2020 Oct 8;11:5072. doi: 10.1038/s41467-020-18759-0 (PMC7544890; doi:10.1038/s41467-020-18759-0)
Supplement: Supplementary file 1 — Supplementary Information [file 41467_2020_18759_MOESM1_ESM.pdf]

Supplementary Information for "Reconstructing the electrical structure of dust storms from locally observed electric field data"

Zhang et al.

Sensor No.	Measured E-field component	Measurement point	Sensor coordinate (in units of m)
s1	E_x	p9	$(0,0,5)$
s2	E_x	p2	$(10,0,5)$
s3	E_x	p1	$(20,0,5)$
s4	E_x	p3	$(0,-10,5)$
s5	E_x	p4	$(0,-5,5)$
s6	E_x	p5	$(0,5,5)$
s7	E_x	p6	$(0,10,5)$
s8	E_y	p3	$(0,-10,5)$
s9	E_y	p4	$(0,-5,5)$
s10	E_y	p9	$(0,0,5)$
s11	E_y	p5	$(0,5,5)$
s12	E_y	p6	$(0,10,5)$
s13	E_y	p2	$(10,0,5)$
s14	E_y	p1	$(20,0,5)$
s15	E_z	p7	$(0,0,1.55)$
s16	E_z	p8	$(0,0,3.35)$
s17	E_z	p9	$(0,0,5)$
s18	E_z	p10	$(0,0,6.95)$
s19	E_z	p11	$(0,0,8.55)$

Supplementary Table 1: **Arrangements of the vibrating-reed electric field mills in the 2017 field observations.** The field observations were performed at the Qingtu Lake Observation Array. A total of 19 vibrating-reed electric field mills (No. s1-s19) were used to measure the number of 19 E-field components (E_x, E_y, and E_z point in the positive directions of x-, y-, and z-axis, respectively) at the measurement points p1-p11.

Supplementary Note 1: Brief description of the vibrating-reed electric field mill

The vibrating-reed electric field mill (VREFM) was developed by Lanzhou University in 2010 (Supplementary Fig. 1). Unlike the traditional rotating vane field mill, the VREFM is based on the vibrating capacitor technique[1,2]. As shown in Supplementary Fig. 2, the vibrating capacitor is composed of an equivalent fixed electrode and a vibrating electrode.

When the vibrating electrode is subjected to a harmonic vibration, the equivalent distance between the two electrodes, $d(t)$, can be written as

$$d(t) = d_0 + \Delta d \sin(\omega t), \qquad \Delta d \ll d_0 \tag{1}$$

where d_0 is the distance between the fixed electrode and the static position of the vibrating electrode, Δd is the amplitude of the vibration, and ω is the frequency of the vibration.

Let $C_0 = \varepsilon_0 \pi r^2/d_0$ and $\Theta = \Delta d/d_0 \ll 1$. Then the capacitance of the vibrating capacitor, C, can be approximately expressed by

$$C = \frac{C_0}{1+\Theta sin(\omega t)} \tag{2}$$

where C_0 is the capacitance of the vibrating capacitor at the static position of the vibrating electrode, and $\varepsilon_0 = 8.85\times10^{-12}$ $C^2N^{-1}m^{-2}$ is the permittivity of air. Let the normal component of the ambient electric field be denoted by $E_n\mathbf{n}$, where $\mathbf{n}$ is the outward normal of the surface of the vibrating electrode (as depicted in Supplementary Figs. 1 and 2), the total induced charge $q(t)$ on the vibrating electrodes is

$$q(t) = -\frac{C_0 d_0 E_n}{1+\Theta \sin(\omega t)} \approx -q_0[1-\Theta \sin(\omega t)] \tag{3}$$

where $q_0 = C_0 d_0 E_n = \varepsilon_0 \pi r^2 E_n$. Due to $\Theta \ll 1$, the direction of the normal component of the electric field, i.e., in the same or opposite direction of $\mathbf{n}$, can be determined by the polarity of q_0. Since the induced electric current on the electrode is $i_d(t) = dq(t)/dt$, we have

$$i_d(t) = q_0 \omega \Theta \cos(\omega t) \tag{4}$$

If the electric current passes through an electric circuit with an electrical resistance of R, the alternating current (AC) voltage is

$$v_d(t) = Rq_0\omega\Theta\cos(\omega t) \tag{5}$$

After rectifying, amplifying and filtering the AC voltage in the signal processing modules, the magnitude of the output (direct current) DC voltage of the VREFM, V_{meas}, can be written as

$$V_{\text{meas}} \sim R\,|q_0|\,\omega\Theta = \varepsilon_0\pi r^2 R\omega\Theta\,|E_n| \tag{6}$$

From Eq. (6), we can see that the value of V_{meas} is directly proportional to $|E_n|$. Consequently, the VREFM can be easily used to measure the electric field component normal to the electrode surface.

In practice, the proportionality constant between the V_{meas} and E_n can be determined by the calibration experiment. As shown in Supplementary Fig. 3a, the VREFM is calibrated using a large parallel-plate electric-field calibrator, where a standard uniform electric field is generated in its central region. Supplementary Fig. 3b shows that, at each applied electric field intensity, the output voltage of the VREFM almost remains constant with a maximum uncertainty of approximately $\pm$ 2.24%. Additionally, there is an excellent linear relationship between the output voltages of the VREFM and the applied electric field intensities, as shown in Supplementary Fig. 3c. In the field observation, the VREFMs were directed along the positive x-, y-, and z-axis to measure the E_x, E_y, and E_z components of the electric field, respectively.

It is worth noting that, when the VREFM is employed in dust events, there is an additional electric current disturbance caused by the charged particles randomly impacting on the vibrating electrode. In contrast to the induced electric circuit that is a periodic signal with a constant frequency of ω, we can find that such a impacting current is a random pulsing disturbance. By properly setting the frequency ω and filtering circuit in the signal processing modules, the random pulsing disturbance can be considerably filtered out.

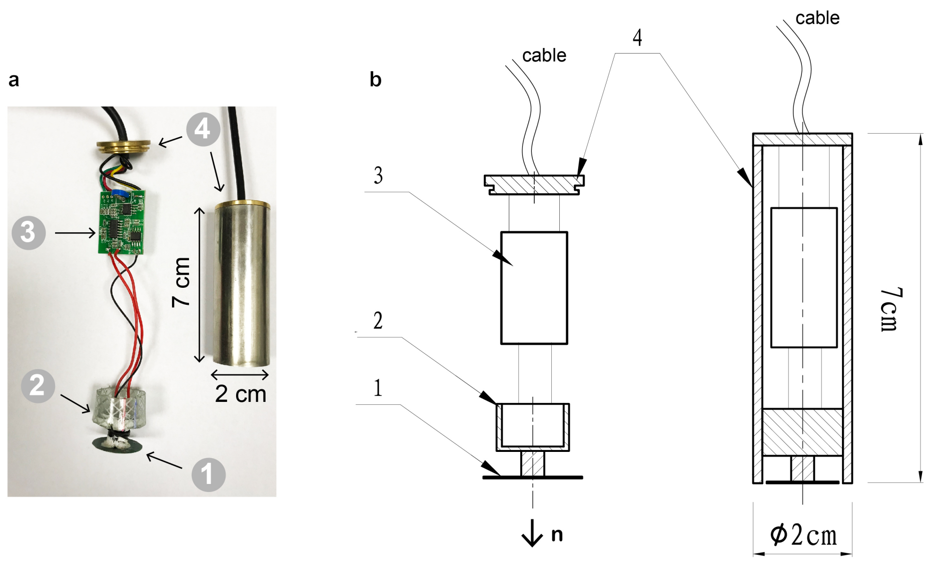

Supplementary Figure 1: **Schematic diagram of the VREFM. a** Exploded view of the VREFM. **b** Sketch of the VREFM assembly. The VREFM consists of four main components: (1) a circular plate with a harmonic vibration is known as the vibrating electrode; (2) a vibrator that drives the vibrating electrode; (3) the signal processing modules that include the current-to-voltage converter, rectification, direct current amplifier, and digital filtering circuits; (4) the shielding hollow cylinder with 7-cm-long, in which the capacitor and the electric circuits are surrounded by the cylinder. Here, **n** is the outward normal of the surface of the vibrating electrode.

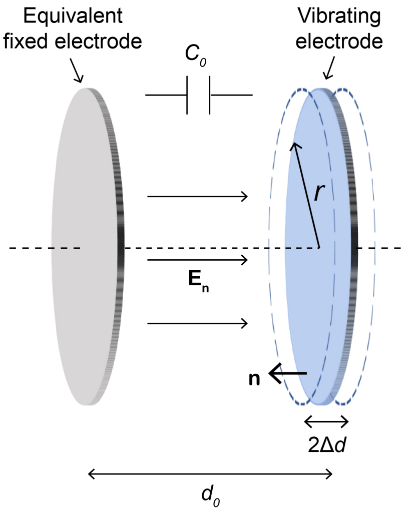

Supplementary Figure 2: **Schematic diagram of the equivalent capacitor of the VREFM.** The dynamic capacitor is equivalent to a fixed electrode parallel to a vibrating electrode. Here, C_0 is the capacitance of the vibrating capacitor at the static position of the vibrating electrode, $\mathbf{E_n}$ is the electric field component normal to the electrode surface, d_0 is the distance between the fixed electrode and the static position of the vibrating electrode, Δd is the amplitude of the vibration, and $\mathbf{n}$ is the outward normal of the surface of the vibrating electrode.

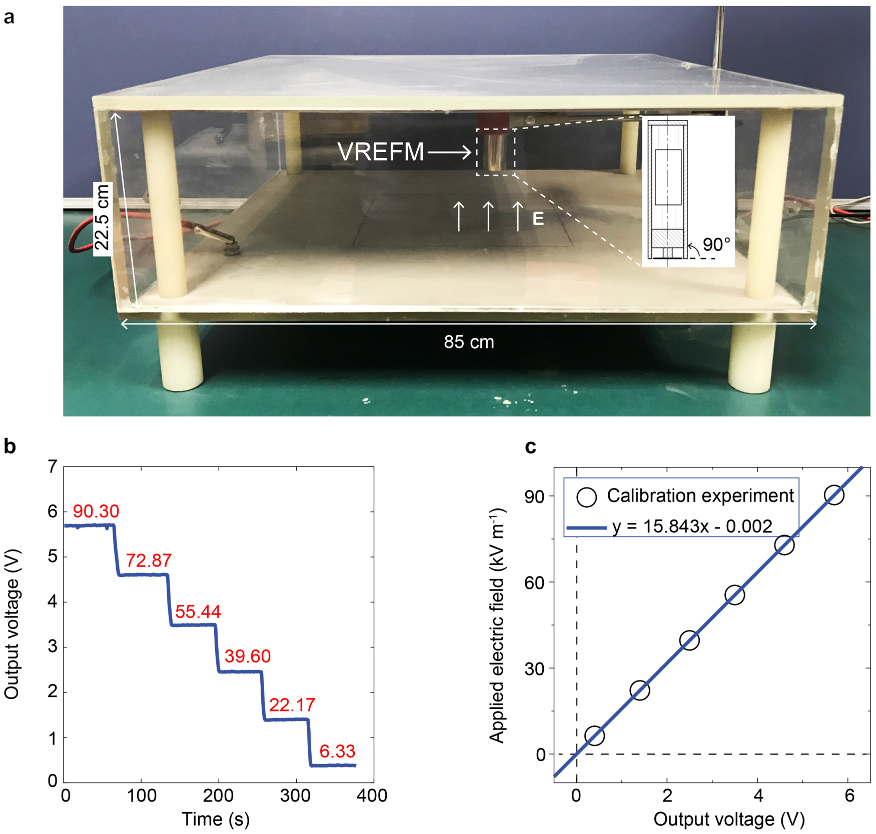

Supplementary Figure 3: **Calibration results of the VREFM in a standard uniform electric field generated by a large parallel-plate electric-field calibrator. a** Set-up of the calibration experiment. The applied uniform electric field **E** is upward-pointing and the outer surface of the VREFM is oriented downward. **b** Time series of the output voltages of the VREFM at six different applied electric field levels (from ~ 6.33 kV m^{-1} to ~ 90.3 kV m^{-1} labeled in red). **c** The significant linear relationships (coefficient of determination $R^2 = 0.999$, p-value < 0.0001) between the output voltages of the VREFM and the magnitudes of the applied electric fields.

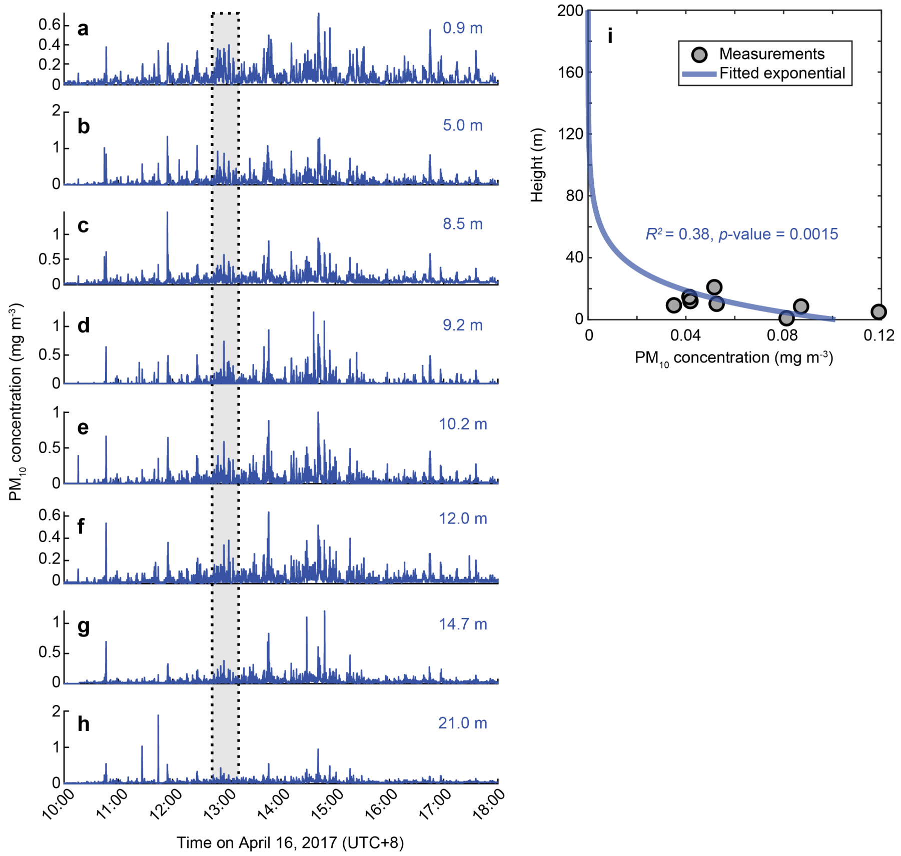

Supplementary Figure 4: **Vertical profile of the PM_{10} concentration for storm #1. a**-**h** Time series of the PM_{10} concentration measured at 0.9-21 m heights (the data for 30 m was not available during storm #1). Here, the grey shaded area represents the relatively steady period of storm #1, whose mean PM_{10} concentration is used to exponentially fit its vertical profile. **i** The fitted vertical profile of PM_{10} concentration for the storm #1, where open circles (○) represent the mean PM_{10} concentration during the shaded period in (**a**-**h**), line is the fitted exponential, and R^2 is the coefficient of determination.

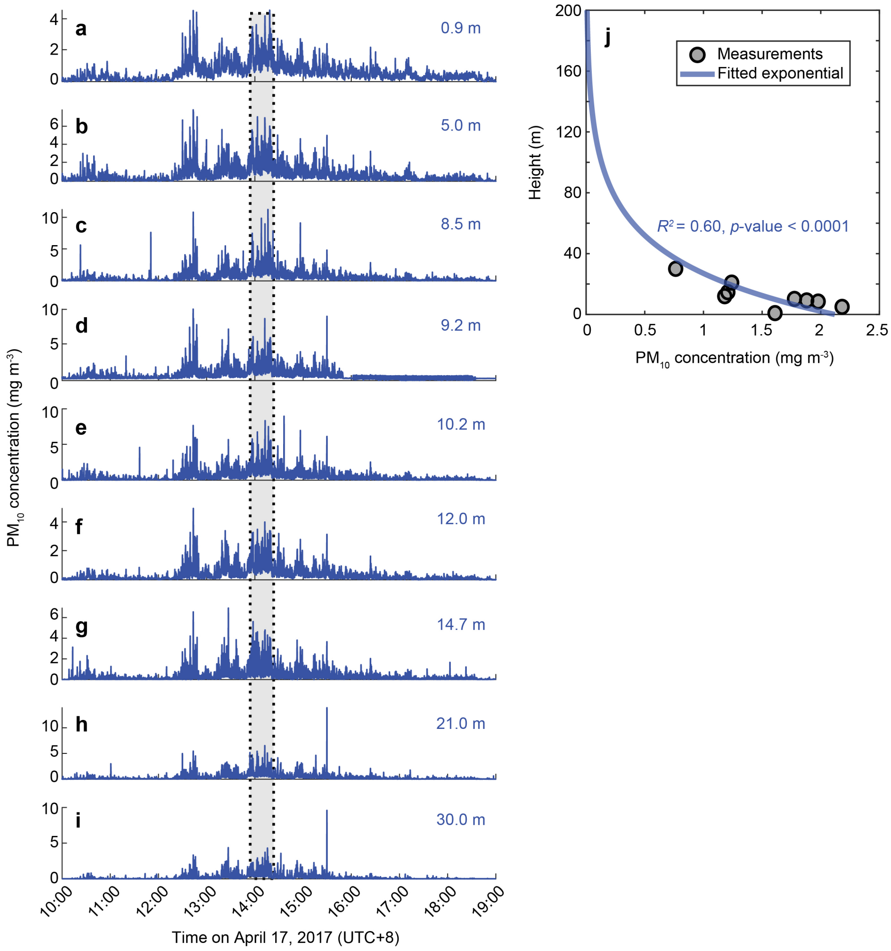

Supplementary Figure 5: **Vertical profile of the PM_{10} concentration for storm #2. a-i** Time series of the PM_{10} concentration measured at 0.9-30 m heights. Here, the grey shaded area represents the relatively steady period of storm #2, whose mean PM_{10} concentration is used to exponentially fit its vertical profile. **j** The fitted vertical profile of PM_{10} concentration for the storm #2, where open circles (○) represent the mean PM_{10} concentration during the shaded period in (**a**-**i**), line is the fitted exponential, and R^2 is the coefficient of determination.

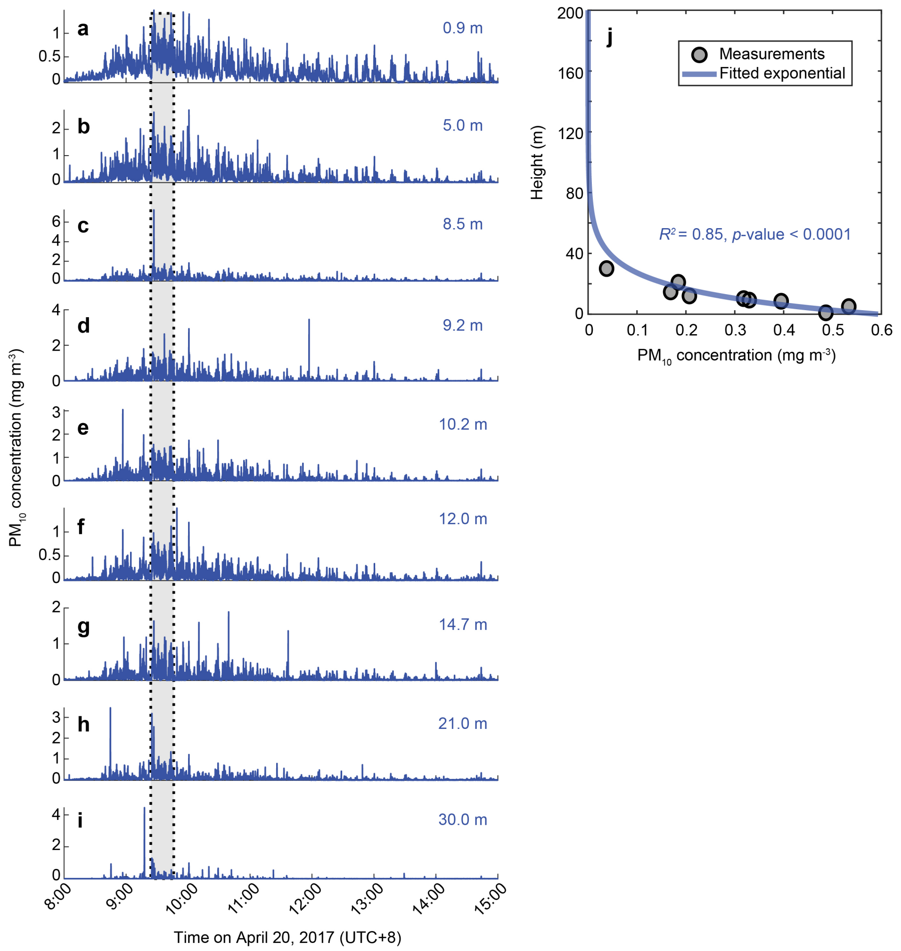

Supplementary Figure 6: **Vertical profile of the PM_{10} concentration for storm #3. a-i** Time series of the PM_{10} concentration measured at 0.9-30 m heights. Here, the grey shaded area represents the relatively steady period of storm #3, whose mean PM_{10} concentration is used to exponentially fit its vertical profile. **j** The fitted vertical profile of PM_{10} concentration for the storm #3, where open circles (○) represent the mean PM_{10} concentration during the shaded period in (**a**-**i**), line is the fitted exponential, and R^2 is the coefficient of determination.

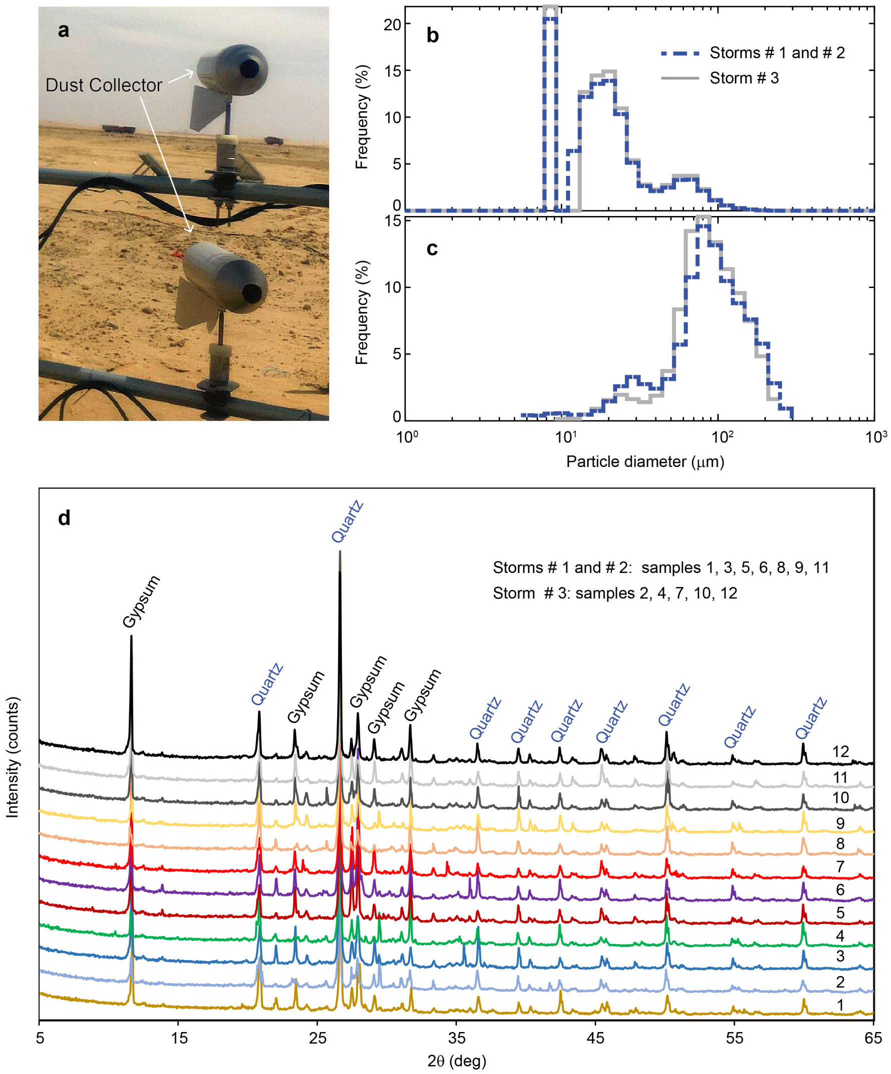

Supplementary Figure 7: **Particle size distributions and mineralogical compositions of the airborne dust particles collected by two dust collectors.** **a** Two dust collectors were mounted on the main tower of the Qingtu Lake Observation Array. **b** The number distributions of the collected dust particles during different periods. **c** The volume distributions of the collected dust particles during different periods. Note that storms #1 and #2 occurred during the first period (blue lines), and storm #3 occurred during the second period (grey lines). **d** The X-ray powder diffraction (XRD) diffractograms of the collected dust samples 1-12. Here, 2θ is the scattering angle. Samples 1, 3, 5, 6, 8, 9, 11 were collected from storms #1 and #2, and the remaining samples were collected from storm #3. The XRD diffractograms are vertically displaced for clarity. It is clear that the XRD diffractograms of the 12 collected dust samples are very similar, and the main mineralogical compositions of the collected dust samples are quartz and gypsum.

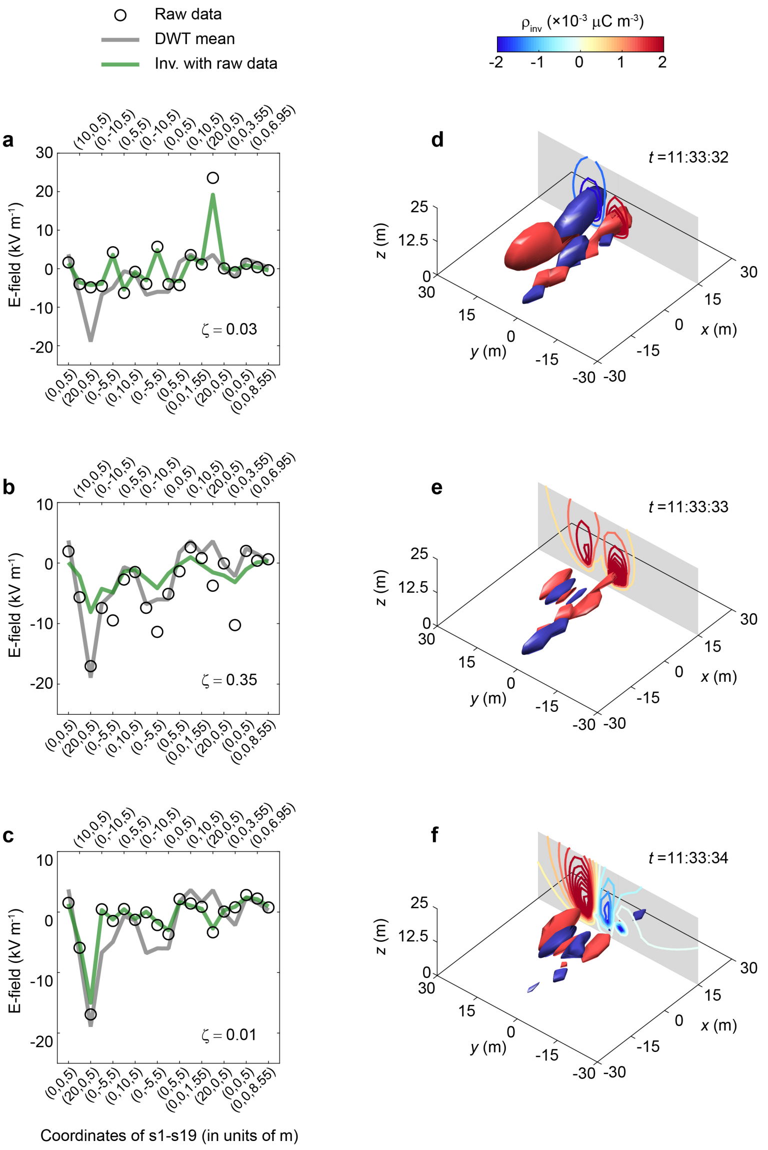

Supplementary Figure 8: **Inversions with instantaneous E-field data of storm #1. a-c** Comparisons of the raw electric field data (Raw data), their discrete wavelet transform mean (DWT mean), and the electric fields predicted by the inversion model (Inv. with raw data) at the positions of sensors s1-s19. Here, ζ is the normalized residual defined in the paper. **d-f** The reconstructed space-charge densities ρ_{inv} correspond to the cases of (**a**-**c**). The isosurfaces are shown at a space-charge density magnitude of 8×10^{-3} μC m^{-3}; the positive surfaces are colored in red, while the negative surfaces are colored in blue. Contourslices at $x = 15$ m are colored based on the space-charge densities. Times t are shown as the local time on April 16, 2017 (UTC+8).

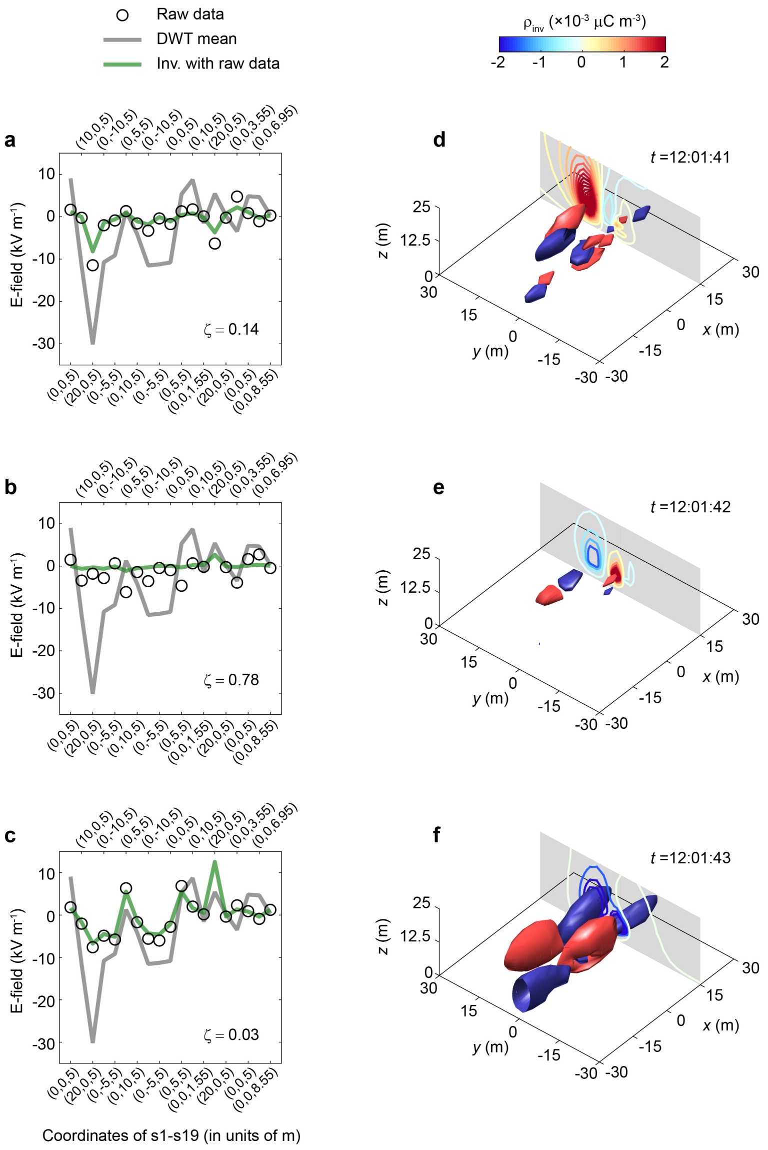

Supplementary Figure 9: **Inversions with instantaneous E-field data of storm #2. a-c** Comparisons of the raw electric field data (Raw data), their discrete wavelet transform mean (DWT mean), and the electric fields predicted by the inversion model (Inv. with raw data) at the positions of sensors s1-s19. Here, ζ is the normalized residual defined in the paper. **d-f** The reconstructed space-charge densities ρ_{inv} correspond to the cases of (**a**-**c**). The isosurfaces are shown at a space-charge density magnitude of 5×10^{-3} μC m^{-3}; the positive surfaces are colored in red, while the negative surfaces are colored in blue. Contourslices at $x = 15$ m are colored based on the space-charge densities. Times t are shown as the local time on April 17, 2017 (UTC+8).

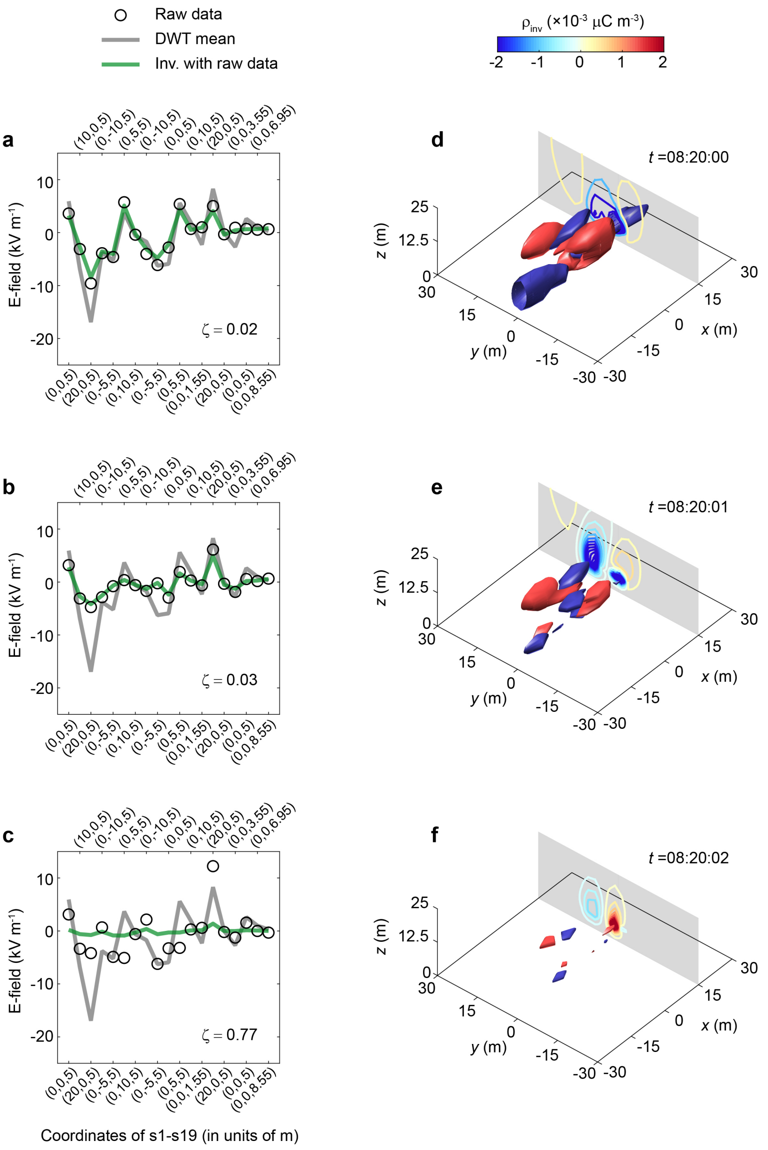

Supplementary Figure 10: **Inversions with instantaneous E-field data of storm #3. a**-**c** Comparisons of the raw electric field data (Raw data), their discrete wavelet transform mean (DWT mean), and the electric fields predicted by the inversion model (Inv. with raw data) at the positions of sensors s1-s19. Here, ζ is the normalized residual defined in the paper. **d**-**f** The reconstructed space-charge densities ρ_{inv} correspond to the cases of (**a**-**c**). The isosurfaces are shown at a space-charge density magnitude of 5×10^{-3} μC m^{-3}; the positive surfaces are colored in red, while the negative surfaces are colored in blue. Contourslices at $x = 15$ m are colored based on the space-charge densities. Times t are shown as the local time on April 20, 2017 (UTC+8).

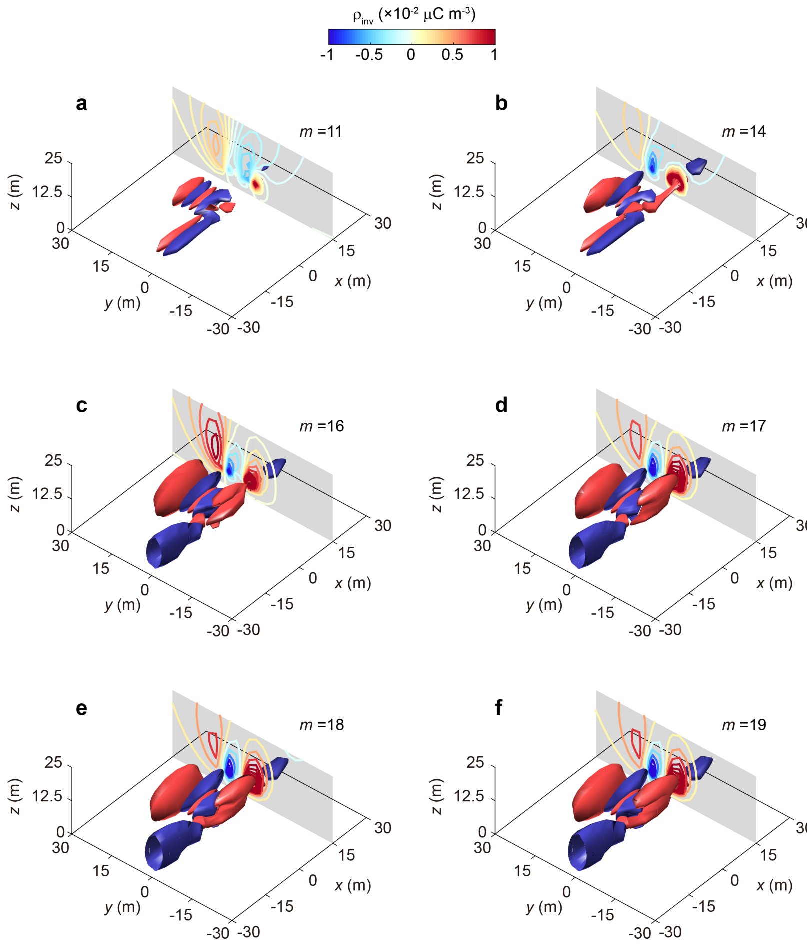

Supplementary Figure 11: **Convergence of the reconstructed space-charge density as the size of subsample increases for storm #1.** **a-f** The 10-trial averaged space-charge densities ρ_{inv} for the subsampling size m varying from 11 to 19. The time point is 13:20:00 on April 16, 2017 (UTC+8). The isosurfaces are shown at a space-charge density magnitude of 2×10^{-2} μC m^{-3}; the positive surfaces are colored in red, while the negative surfaces are colored in blue. Contourslices at $x = 15$ m are colored based on the space-charge densities.

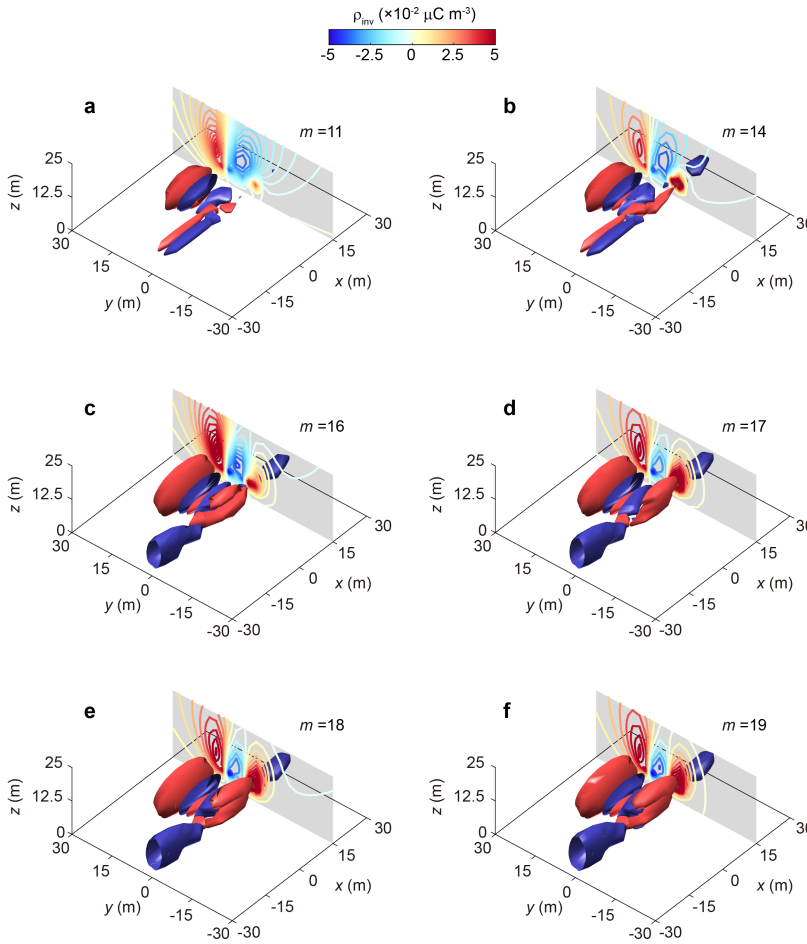

Supplementary Figure 12: **Convergence of the reconstructed space-charge density as the size of subsample increases for storm #2. a-f** The 10-trial averaged space-charge densities ρ_{inv} for the subsampling size m varying from 11 to 19. The time point is 14:10:00 on April 17, 2017 (UTC+8). The isosurfaces are shown at a space-charge density magnitude of 9×10^{-2} μC m^{-3}; the positive surfaces are colored in red, while the negative surfaces are colored in blue. Contourslices at $x = 15$ m are colored based on the space-charge densities.

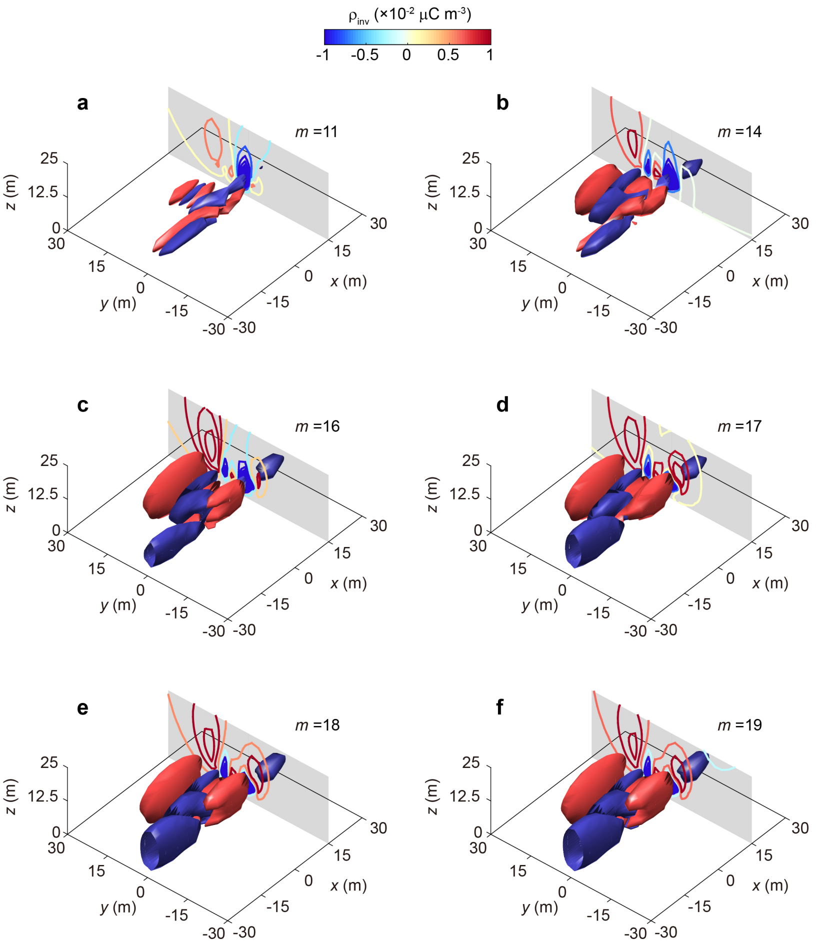

Supplementary Figure 13: **Convergence of the reconstructed space-charge density as the size of subsample increases for storm #3. a-f** The 10-trial averaged space-charge densities ρ_{inv} for the subsampling size m varying from 11 to 19. The time point is 09:35:00 on April 20, 2017 (UTC+8). The isosurfaces are shown at a space-charge density magnitude of 4×10^{-2} μC m^{-3}; the positive surfaces are colored in red, while the negative surfaces are colored in blue. Contourslices at $x = 15$ m are colored based on the space-charge densities.

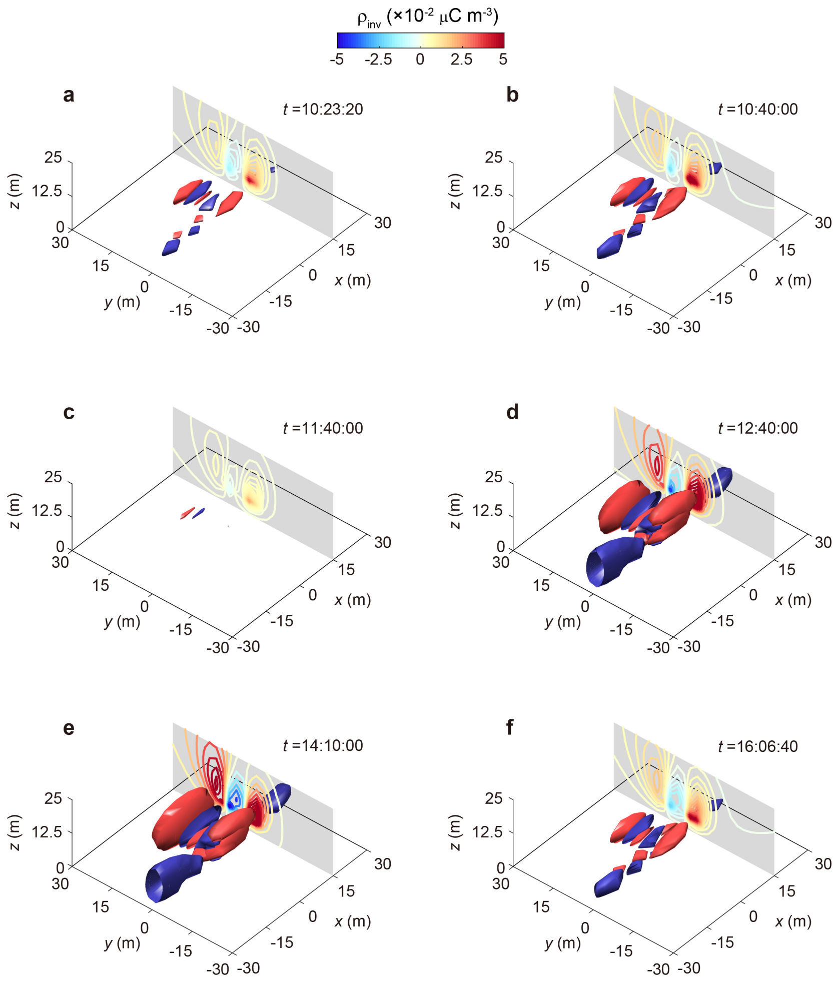

Supplementary Figure 14: **Evolution of the 3D structure of the space-charge densities during storm #2. a-f** The reconstructed space-charge densities ρ_{inv} at different stages of the observed dust storm. The isosurfaces are shown at a space-charge density magnitude of 9×10^{-2} μC m^{-3}; the positive surfaces are colored in red, while the negative surfaces are colored in blue. Times t are shown as the local time on April 17, 2017 (UTC+8). Contourslices at $x = 15$ m are colored based on the space-charge densities.

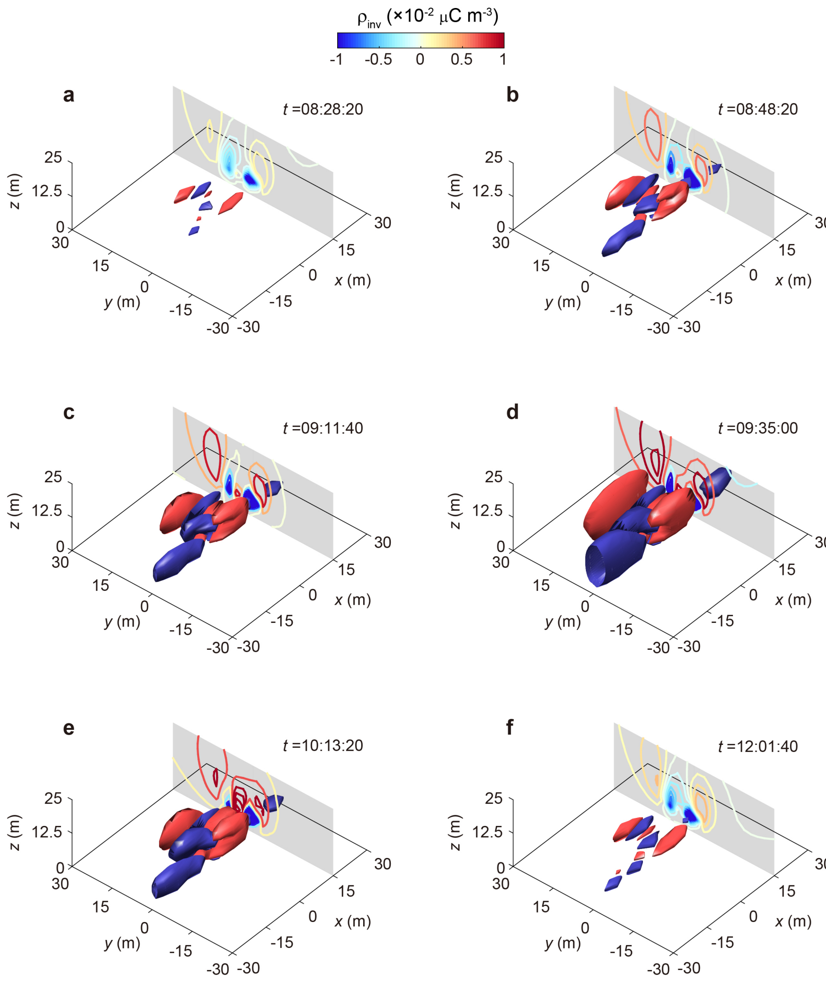

Supplementary Figure 15: **Evolution of the 3D structure of the space-charge densities during storm #3. a-f** The reconstructed space-charge densities ρ_{inv} at different stages of the observed dust storm. The isosurfaces are shown at a space-charge density magnitude of 4×10^{-2} μC m^{-3}; the positive surfaces are colored in red, while the negative surfaces are colored in blue. Times t are shown as the local time on April 20, 2017 (UTC+8). Contourslices at $x = 15$ m are colored based on the space-charge densities.

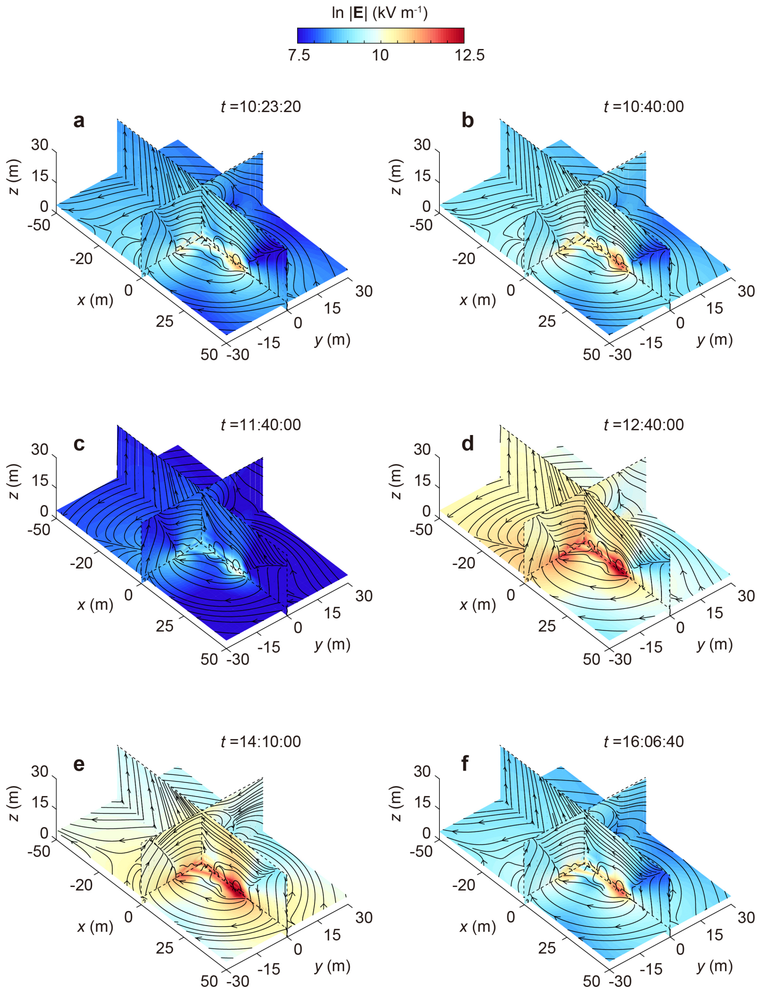

Supplementary Figure 16: **Evolution of the 3D structure of the E-fields during storm #2. a-f** E-field predicted from the reconstructed space-charge densities at different stages of the observed storm. Slices at $x = 0$ m, $y = 0$ m, $z = 4$ m are colored based on the log-magnitude of the 3D E-field, $\ln|\mathbf{E}|$. Times t are shown as the local time on April 17, 2017 (UTC+8). Lines represent the E-field lines.

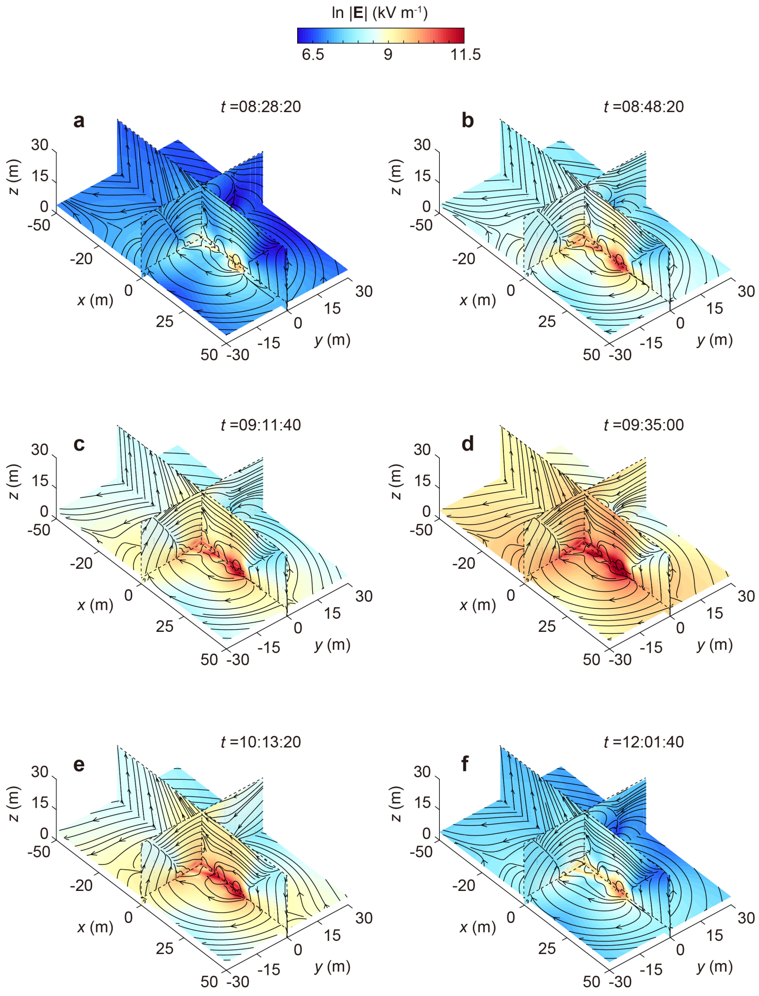

Supplementary Figure 17: **Evolution of the 3D structure of the E-fields during storm #3. a-f** Slices at $x = 0$ m, $y = 0$ m, $z = 4$ m are colored based on the log-magnitude of the 3D E-field, $\ln|\mathbf{E}|$. Times t are shown as the local time on April 20, 2017 (UTC+8). Lines represent the E-field lines.

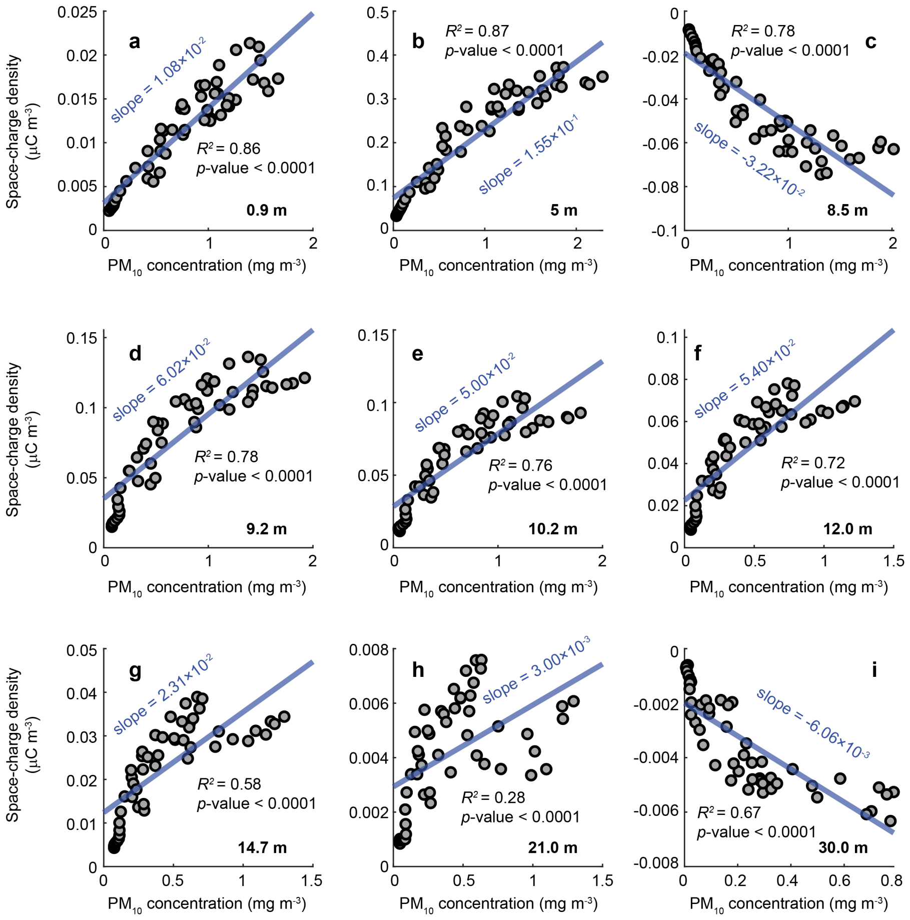

Supplementary Figure 18: **Significant linear relationships between the PM_{10} concentrations and space-charge densities for storm #2. a-i** Linear relationships at the heights from 0.9 m to 30 m. Here, symbols denote the reconstructed space-charge density vs. the 2^9 s time-varying mean of the measured PM_{10} concentration (extracted by the discrete wavelet transform), and lines denote linear regressions (coefficient of determination R^2 and p-value are shown). For these data, the ambient temperature and relative humidity are in the range of 20.2 ± 0.5 °C and $20.1 \pm 0.9\%$, respectively.

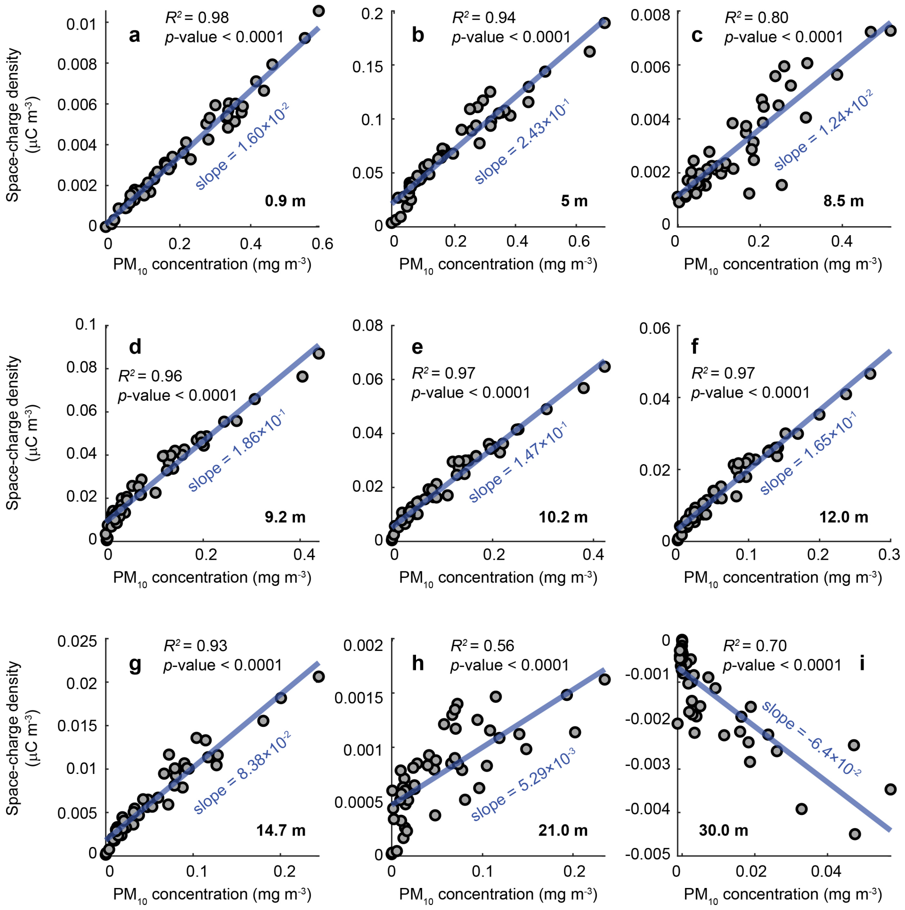

Supplementary Figure 19: **Significant linear relationships between the PM_{10} concentrations and space-charge densities for storm #3. a-i** Linear relationships at the heights from 0.9 m to 30 m. Here, symbols denote the reconstructed space-charge density vs. the 2^9 s time-varying mean of the measured PM_{10} concentration (extracted by the discrete wavelet transform), and lines denote linear regressions (coefficient of determination R^2 and p-value are shown). For these data, the ambient temperature and relative humidity are in the range of 9.0 ± 1.4 °C and $15.6 \pm 2.5\%$, respectively.

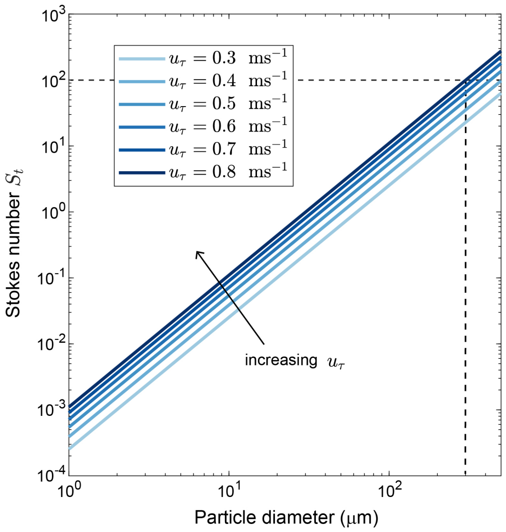

Supplementary Figure 20: **Estimated Stokes number as a function of particle diameter at different wind shear velocities.** The vertical dashed line represents the maximum diameter of the dust particles collected at 5 m height (see Supplementary Fig. 7). For the observed storms #1-#3, the maximum wind shear velocity u_τ is approximately 0.72 m s^{-1}.

Supplementary References

[1] Rohwerder, M. & Turcu, F. High-resolution Kelvin probe microscopy in corrosion science: scanning Kelvin probe force microscopy (SKPFM) versus classical scanning Kelvin probe (SKP). *Electrochim. Acta* **53,** 290-299 (2007).

[2] Kobayashi, T., Oyama, S., Akahashi, M., Maeda, R. & Itoh, T. Microelectromechanical systems-based electrostatic field sensor using $Pb(Zr,Ti)O_3$ thin films. *Jpn. J. Appl. Phys.* **47,** 7533–7536 (2008).
